# Administration of a single dose of lithium ameliorates rhabdomyolysis-associated acute kidney injury in rats

**DOI:** 10.1371/journal.pone.0281679

**Published:** 2023-02-16

**Authors:** Maria Heloisa Massola Shimizu, Rildo Aparecido Volpini, Ana Carolina de Bragança, Mariana Moura Nascimento, Desiree Rita Denelle Bernardo, Antonio Carlos Seguro, Daniele Canale

**Affiliations:** 1 Laboratorio de Investigacao Medica 12 (LIM12), Faculdade de Medicina, Universidade de São Paulo, São Paulo, Brazil; 2 Laboratorio de Investigacao Medica 12 (LIM12), Hospital das Clinicas HCFMUSP, Faculdade de Medicina, Universidade de São Paulo, São Paulo, Brazil; Uniformed Services University, UNITED STATES

## Abstract

Rhabdomyolysis is characterized by muscle damage and leads to acute kidney injury (AKI). Clinical and experimental studies suggest that glycogen synthase kinase 3β (GSK3β) inhibition protects against AKI basically through its critical role in tubular epithelial cell apoptosis, inflammation and fibrosis. Treatment with a single dose of lithium, an inhibitor of GSK3β, accelerated recovery of renal function in cisplatin and ischemic/reperfusion-induced AKI models. We aimed to evaluate the efficacy of a single dose of lithium in the treatment of rhabdomyolysis-induced AKI. Male Wistar rats were allocated to four groups: Sham, received saline 0.9% intraperitoneally (IP); lithium (Li), received a single IP injection of lithium chloride (LiCl) 80 mg/kg body weight (BW); glycerol (Gly), received a single dose of glycerol 50% 5 mL/kg BW intramuscular (IM); glycerol plus lithium (Gly+Li), received a single dose of glycerol 50% IM plus LiCl IP injected 2 hours after glycerol administration. After 24 hours, we performed inulin clearance experiments and collected blood / kidney / muscle samples. Gly rats exhibited renal function impairment accompanied by kidney injury, inflammation and alterations in signaling pathways for apoptosis and redox state balance. Gly+Li rats showed a remarkable improvement in renal function as well as kidney injury score, diminished CPK levels and an overstated decrease of renal and muscle GSK3β protein expression. Furthermore, administration of lithium lowered the amount of macrophage infiltrate, reduced NFκB and caspase renal protein expression and increased the antioxidant component MnSOD. Lithium treatment attenuated renal dysfunction in rhabdomyolysis-associated AKI by improving inulin clearance and reducing CPK levels, inflammation, apoptosis and oxidative stress. These therapeutic effects were due to the inhibition of GSK3β and possibly associated with a decrease in muscle injury.

## Introduction

Rhabdomyolysis is a clinical syndrome characterized by the destruction of skeletal muscle after direct injury with subsequent outflow of its intracellular compounds into the bloodstream, leading to systemic complications [1]. Acute kidney injury (AKI) develops in 10 to 55% of patients and it is the most severe complication of rhabdomyolysis [2, 3]. Rhabdomyolysis-associated AKI promotes renal structural alterations, such as glomerulosclerosis and fibrosis, and is frequently related to a poor outcome [3].

Rhabdomyolysis-associated AKI may be induced in a murine experimental model by a single intramuscular injection of glycerol [4–6]. Glycerol-induced AKI is marked by myoglobinuria, which is the main cause of renal damage in rhabdomyolysis. Besides myoglobinuria, enhanced renal vasoconstriction, ischemic tubular injury, tubular obstruction and the direct toxicity of heme protein are also involved in the progression of glycerol-induced renal failure [6–8]. The specific mechanisms by which rhabdomyolysis leads to the impairment of renal function are still unclear [9]. Nevertheless, experimental evidences suggest that cellular release of myoglobin results in uncontrolled generation of reactive oxygen species (ROS), triggering inflammation events and apoptosis through the overactivation of NFkB and mitochondrial disruption, respectively [5, 10, 11]. Moreover, it has been demonstrated that myoglobin itself may display increased lipid peroxidation and production of isoprostanes [9].

Some studies have demonstrated that prompt treatment is commonly related to a better prognosis in cases of rhabdomyolysis-associated AKI [12, 13]. Gois *et al*. reported that treatment with allopurinol, a xanthine oxidase inhibitor, diminished oxidative stress, inhibited apoptosis and reduced inflammatory cells infiltration in glycerol-induced AKI experimental model [14]. In addition, it has been suggested that glycogen synthase kinase 3β (GSK3β) inhibition may be a newly therapeutic strategy to improve renal outcomes after AKI. Treatment with a single dose of lithium, an inhibitor of GSK3β, accelerated recovery of renal function in cisplatin and ischemic/reperfusion-induced AKI models [15]. In the light of the aforementioned, the aim of this study was to evaluate the efficacy of a single dose of lithium in the treatment of rhabdomyolysis-induced AKI in rats.

## Materials and methods

We conducted all the experimental procedures in strict conformity with our local institutional guidelines (CEUA-HCFMUSP, process no. 1837/2022) and with well-established international standards for manipulation and care of laboratory animals (Guide for the Care and Use of Laboratory Animals–NCBI–NIH). We performed all surgeries under appropriate anesthesia and all efforts were made to minimize suffering.

### Animals and experimental protocol

Male Wistar rats (*Rattus novergicus*) weighing 180–200 g were obtained from the animal facilities of the University of São Paulo–Institute of Biomedical Sciences. We kept our animals at controlled temperature (23±1°C) with a light/dark cycle of 12/12h in standard cages with *ad libitum* access to tap water and commercial rodent chow (Nuvilab, PR, Brazil). Rats were allocated to four groups: Sham (C, n = 7), received saline 0.9% (5 mg/kg body weight) intraperitoneally (IP); Lithium (Li, n = 7), received a single IP injection of Lithium Chloride (LiCl, 80 mg/kg body weight, MilliporeSigma, MA, USA); Glycerol (Gly, n = 8), received a single dose of glycerol 50% (5 mL/kg body weight, Sigma-Aldrich, MO, USA) intramuscular (IM); and Glycerol plus Lithium (Gly+Li, n = 8), received a single dose of glycerol 50% IM plus LiCl IP injected 2 hours after glycerol administration. We submitted the animals to the inhalational anesthetic isoflurane (1mL/1mL; Cristália, SP, Brazil) for approximately 3 minutes before saline or glycerol infusion. The doses and the time between injections of LiCl and glycerol were based on previous studies [14–16]. In addition, as the human model of severe rhabdomyolysis requires prompt treatment, we chose to administer lithium 2 hours after the glycerol injection in order to reproduce clinical conditions found in the hospital daily routine.

### Glomerular filtration rate (GFR) and hemodynamic studies

To determine glomerular filtration rate (GFR), we performed inulin clearance studies after 24 hours of saline or glycerol administration. We anesthetized the animals with sodium thiopental (50 mg/Kg BW) and then we cannulated the trachea with a PE-240 catheter for spontaneous breathing. The jugular vein was cannulated with PE-60 catheter for infusion of inulin and fluids. To monitor mean arterial pressure (MAP, mmHg) and collect blood samples, the right femoral artery was catheterized with a PE-50 catheter. We assessed MAP with a data acquisition system (MP100; Biopac Systems, CA, USA). To collect urine samples, we cannulated the bladder with a PE-240 catheter by suprapubic incision. After the surgical procedure, a loading dose of inulin (100 mg/Kg BW diluted in 1 mL of 0.9% saline) was administered through the jugular vein. A constant infusion of inulin (10 mg/Kg BW) was started and continued at 0.04 mL/min throughout the whole experiment. We collected three urine samples at 30-min intervals. Blood samples were obtained at the beginning and at the end of the experiment. Inulin clearance values represent the mean of three periods. Plasma and urinary inulin were determined by the anthrone method and the GFR data were expressed as mL/min/100g BW. To measure renal blood flow (RBF, mL/min), we made a median incision and dissected the left renal pedicle for isolating the renal artery. An ultrasonic flow probe was placed around the exposed renal artery, and RBF was measured with an ultrasonic flow meter (T402; Transonic Systems, MD, USA). We divided blood pressure by RBF to calculate renal vascular resistance (RVR, mmHg/mL/min).

### Evaluation of plasma levels of creatinine phosphokinase (CPK)

To assess plasma levels of CPK, we collected blood samples after the clearance studies. We determined plasma levels of CPK by colorimetric assay (Labtest Diagnóstica, MG, Brazil). The detection system and the quantification followed the protocol described by the manufacturer.

### Tissue sample preparation

Still under anesthesia and after the clearance experiment and blood sample collection, we perfused kidneys with phosphate-buffered solution (PBS, pH 7.4) until the death of the animal by exsanguination. We froze the right kidneys and fragments of muscle from the hind legs in liquid nitrogen and stored at -80ºC for western blotting. The left kidneys were removed and a fragment of the renal tissue was fixed in methacarn solution (60% methanol, 30% chloroform, 10% glacial acetic acid) for 24 h and replaced by 70% alcohol thereafter. The kidney blocks were embedded in paraffin and cut into 4-μm sections for histological and Immunohistochemical (IHC) studies.

### Total protein isolation

Kidney and muscle samples were homogenized in ice-cold isolation solution (200 mM mannitol, 80 mM HEPES and 41 mM KOH, pH 7.5) containing a protease inhibitor cocktail (Sigma Chemical Company, MO, USA) in a homogenizer (Tissue Master TM125, Omni International, GA, USA). Homogenates were centrifuged at 4,000 x rpm for 30 min at 4°C to remove nuclei and cell debris. Supernatants were isolated and protein was quantified by Bradford assay (Bio-Rad Laboratories, Hercules, CA).

### Western blot assays

For western blot analysis, 100 μg of total kidney or muscle protein were separated on SDS-polyacrylamide minigels by electrophoresis [17]. After transfer by electroelution to PVDF membranes (GE Healthcare Limited, Little Chalfont, UK), blots were blocked for 1h with 5% nonfat milk in Tris-buffered saline solution. Blots were then incubated with the primary antibody for anti-GSK3β (1:500; Cell Signaling, MA, USA), anti-NFκB p65 (1:200; Santa Cruz Biotechnology, TX, EUA), anti-caspase-3 (1:500; Cell Signaling, MA, USA) and anti-MnSOD (1:1,000; Cayman Chemicals, MI, USA). The labeling was visualized with a horseradish peroxidase-conjugated secondary antibody (anti-rabbit or anti-mouse, both 1:2,000; Sigma Chemical, MO, USA) and enhanced chemiluminescence (ECL) detection (GE Healthcare Limited, Little Chalfont, UK). Kidney and muscle protein levels were further analyzed with a gel documentation system (Alliance 4.2; Uvitec, Cambridge, UK) and the software Image J for Windows (Image J-NIH Image). We used densitometry to quantitatively analyze the protein levels, normalizing the bands to β-actin expression (1,2000; anti β-actin, Sigma Chemical, MO, USA).

### Light microscopy

Four-mm histological sections of kidney tissue were stained with hematoxylin-eosin (HE) and examined under light microscope. For the evaluation of renal damage, 40–60 grid fields (x400 magnification) measuring 0.245 mm^2^ were evaluated by graded scores according to the following criteria: (0), less than 5% of the field showing tubular epithelial swelling, vacuolar degeneration, necrosis, and desquamation; (I), 5–25% of the field presenting renal lesions; (II), involvement of 25–50% with renal damage; (III), 50–75% of damaged area; and (IV), more than 75% of the grid field presenting renal lesions. After that, we calculated the mean score for each rat and evaluated the mean score for each group [18, 19]. To minimize bias during the morphometric analysis, the observer was blinded to the treatment groups.

### Immunohistochemical analysis

Immunohistochemistry was performed on 4-μm paraffinized kidney sections mounted on 2% silane-coated glass slides. We used a mouse monoclonal antibody to CD68 (1:100; AbD Serotec, Oxford, UK) as a marker of macrophages. We subjected the kidney tissue sections to IHC reaction according to the protocol of the primary antibody. Reaction products were detected by avidin-biotin-peroxidase (Vector Laboratories, Burlingame, CA) and the staining was developed with 3,3-diaminobenzidine (Sigma, St. Louis, MO) in the presence of hydrogen peroxide. Counterstaining was with Harris’ hematoxylin. We analyzed 30–40 renal cortex fields (0.09 mm^2^) to evaluate the immunoreactions. The volume ratios of positive areas of renal tissue (%), determined by the color limit, were obtained by ZEN image analyzer software (Carl Zeiss, Munich, Germany) on a computer coupled to a microscope (Carl Zeiss Axioskop 40) and a digital camera [20, 21]. To minimize bias during the IHC analysis, the observer was blinded to the treatment groups.

### Statistical analysis

All data were expressed as mean ± SEM (standard error of the mean). Differences among groups were analyzed with GraphPad Prism 5.0 software (GraphPad Software, CA, USA) by one-way analysis of variance followed by the Student-Newman-Keuls test. Values of p < 0.05 were considered statistically significant.

## Results

At the end of the experimental protocol, glycerol-injected rats presented a significantly impaired renal function compared to Sham rats. Gly+Li group showed a recovery of GFR compared to Sham and Li groups (Table 1), indicating that treatment with lithium was effective in restoring renal function in a glycerol-induced AKI murine model. It is worth noting that treatment with lithium alone did not alter renal function. We did not observe any differences in MAP, RBF and RVR among the groups (Table 1).

**Table 1:** Renal function and hemodynamic parameters evaluated after the 24h protocol.

	Sham (n = 7)	Li (n = 7)	Gly (n = 8)	Gly+Li (n = 8)
GFR (mL/min/100g)	1.01±0.07	0.86±0.08	0.36±0.06^a^^d^	0.86±0.06^g^
MAP (mmHg)	121±1	121±6	127±2	115±4
RBF (mL/min)	6.44±0.15	6.65±0.23	6.22±0.14	6.30±0.24
RVR (mmHg/mL/min)	18.85±0.43	16.40±1.45	20.46±0.64	18.63±1.51

Sham; Li, lithium; Gly, glycerol; Gly+Li, glycerol + lithium. GFR, glomerular filtration rate; MAP, mean arterial pressure; RBF, renal blood flow; and RVR, renal vascular resistance. Values are means ± SEM.

^a^p < 0.001 vs Sham;

^d^p < 0.001 vs Li;

^g^p < 0.001 vs. Gly.

**Fig 1:**
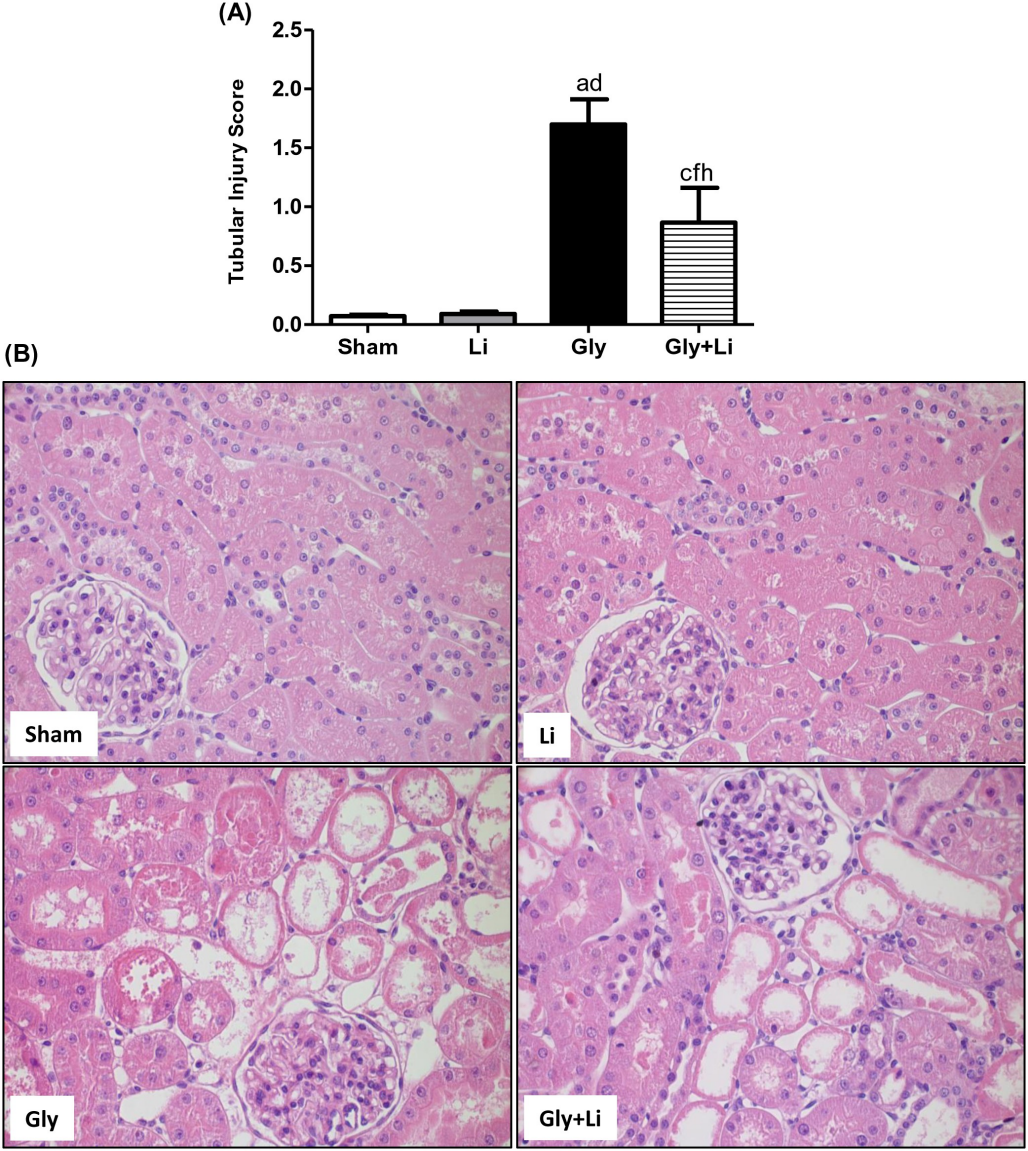
Tubular injury score in the renal cortex evaluated at the end of the 24h protocol. (A) Bar graph of tubular injury score values. (B) Representative photomicrographs of renal histological changes from a Sham, Li, Gly and Gly+Li rat (x400). Values are means ± SEM. ^a^p<0.001, ^c^p<0.05 vs. Sham; ^d^p<0.001, ^f^p<0.05 vs. Li; ^h^p<0.01 vs. Gly. Li, lithium; Gly, glycerol; Gly+Li, glycerol + lithium.

**Fig 2:**
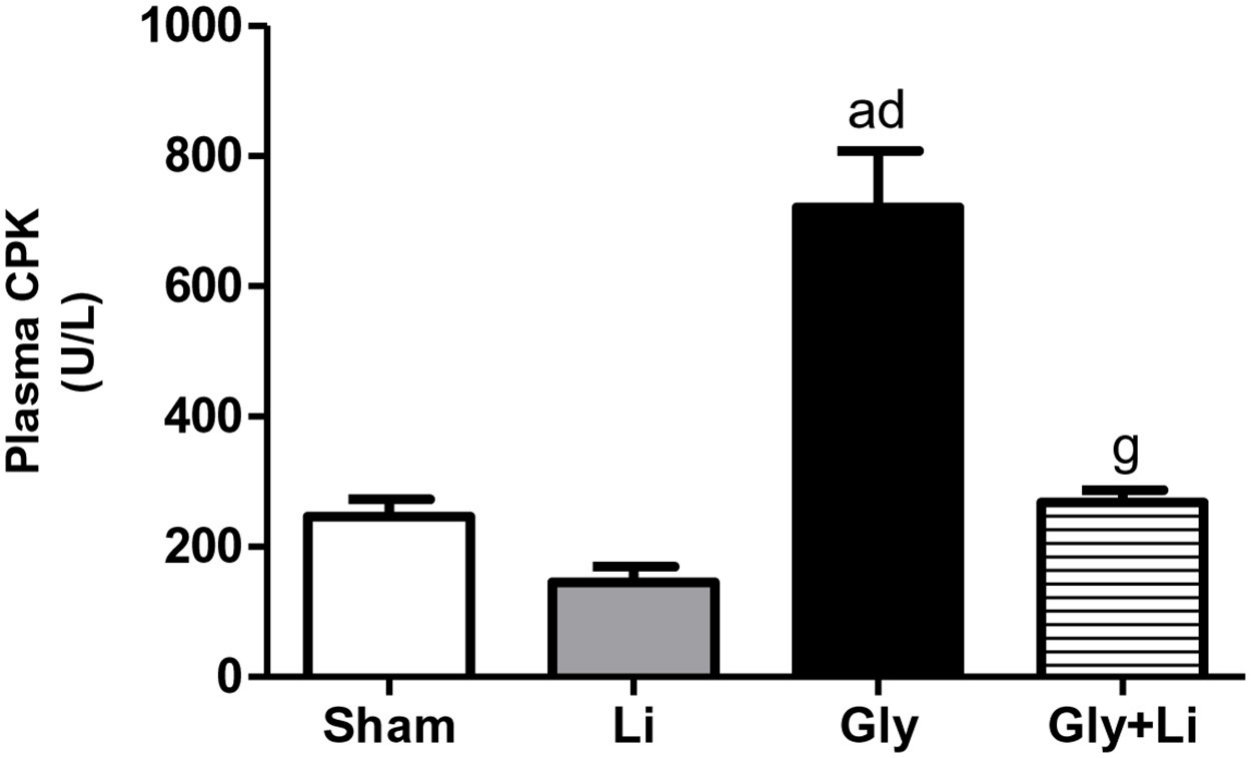
Plasma concentration of Creatine Phosphokinase (CPK) evaluated at the end of the 24h protocol in Sham, Li, Gly and Gly+Li rats. Values are means ± SEM. ^a^p < 0.001 vs Sham; ^d^p < 0.001 vs Li; ^g^p < 0.001 vs. Gly. Li, lithium; Gly, glycerol; Gly+Li, glycerol + lithium.

As expected, histological findings showed characteristic alterations of acute tubular necrosis in the renal cortex of Gly group compared to Sham group, confirming the intense renal impairment in that group. We observed tubular cell necrosis, focal areas of denuded basement membrane, flattening of proximal tubular cells with brush border loss and tubular atrophy or dilatation in the renal cortex of Gly rats. Tubular injury score was clearly attenuated in the Gly+Li group compared to Gly group, corroborating the improvement in renal function by lithium treatment. Rats that received treatment with lithium alone did not present alteration in this parameter (Fig 1). To evaluate the effects of lithium treatment on muscular protection, we determined plasma levels of CPK. Creatinine phosphokinase was markedly increased 24 hours after glycerol administration in Gly group compared to Sham group and its concentration was unchanged in rats treated with lithium alone. However, Gly+Li group exhibited adequate plasma levels of CPK, suggesting that lithium administration might have recovered muscular damage (Fig 2).

**Fig 3:**
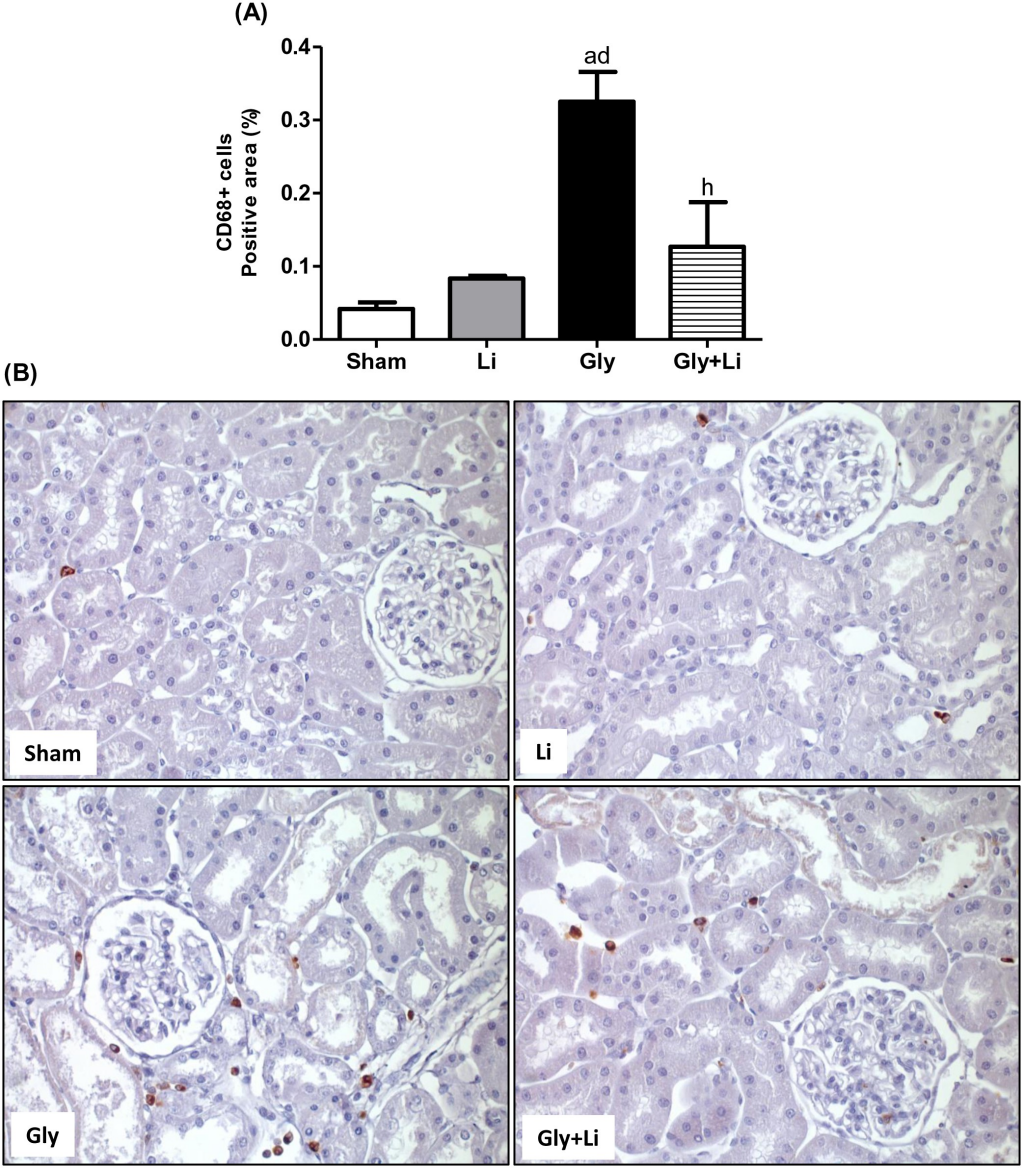
Immunohistochemical analysis for CD68+ cells expression in rat kidney tissue. (A) Bar graph of CD68+ cells expression values. (B) Representative photomicrographs of immunostaining for CD68+ cells in the renal cortex from a Sham, Li, Gly and Gly+Li rat (x400). Values are means ± SEM. ^a^p<0.001 vs. Sham; ^d^p<0.001 vs. Li; ^h^p<0.01 vs. Gly. Li, lithium; Gly, glycerol; Gly+Li, glycerol + lithium.

**Fig 4:**
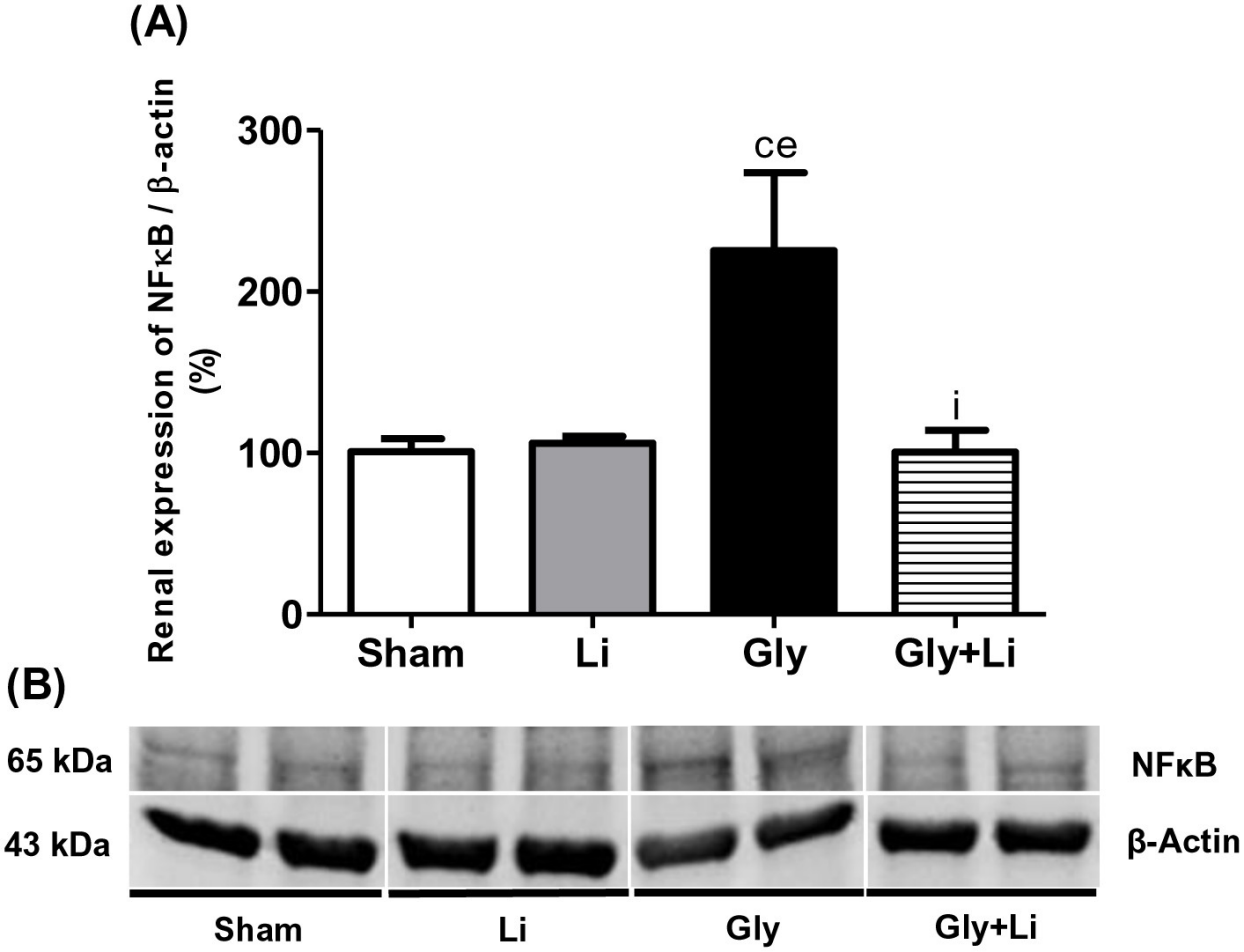
Semiquantitative immunoblotting for NFκB expression in rat kidney tissue. (A) NFκB densitometric analysis of kidney samples from Sham, Li, Gly and Gly+Li rats. (B) Representative immunoblots which reacted with anti-NFκB revealing bands of 65kDa. Values are means ± SEM. ^c^p<0.05 vs. Sham; ^e^p<0.01 vs. Li; ^i^p<0.05 vs. Gly. Li, lithium; Gly, glycerol; Gly+Li, glycerol + lithium.

To evaluate the pro-inflammatory response, we assessed the renal amount of CD68+ cells (macrophages) and the renal protein expression of NFκB p65 subunit. As shown in Fig 3, we found a higher expression of CD68+ cells in the renal cortex of Gly group compared to the other groups. Lithium treatment restored cortical macrophage infiltration in the Gly+Li group compared to Sham and Li groups. Likewise, we observed an increased protein expression of p65 NFκB subunit in the Gly group compared to the Sham and Li groups. Treatment with lithium improved the renal protein expression of p65 NFκB subunit in Gly+Li group compared to Gly group (Fig 4 and S1 Fig). Hence, our results strengthen the importance of lithium administration on the modulation of renal inflammation.

**Fig 5:**
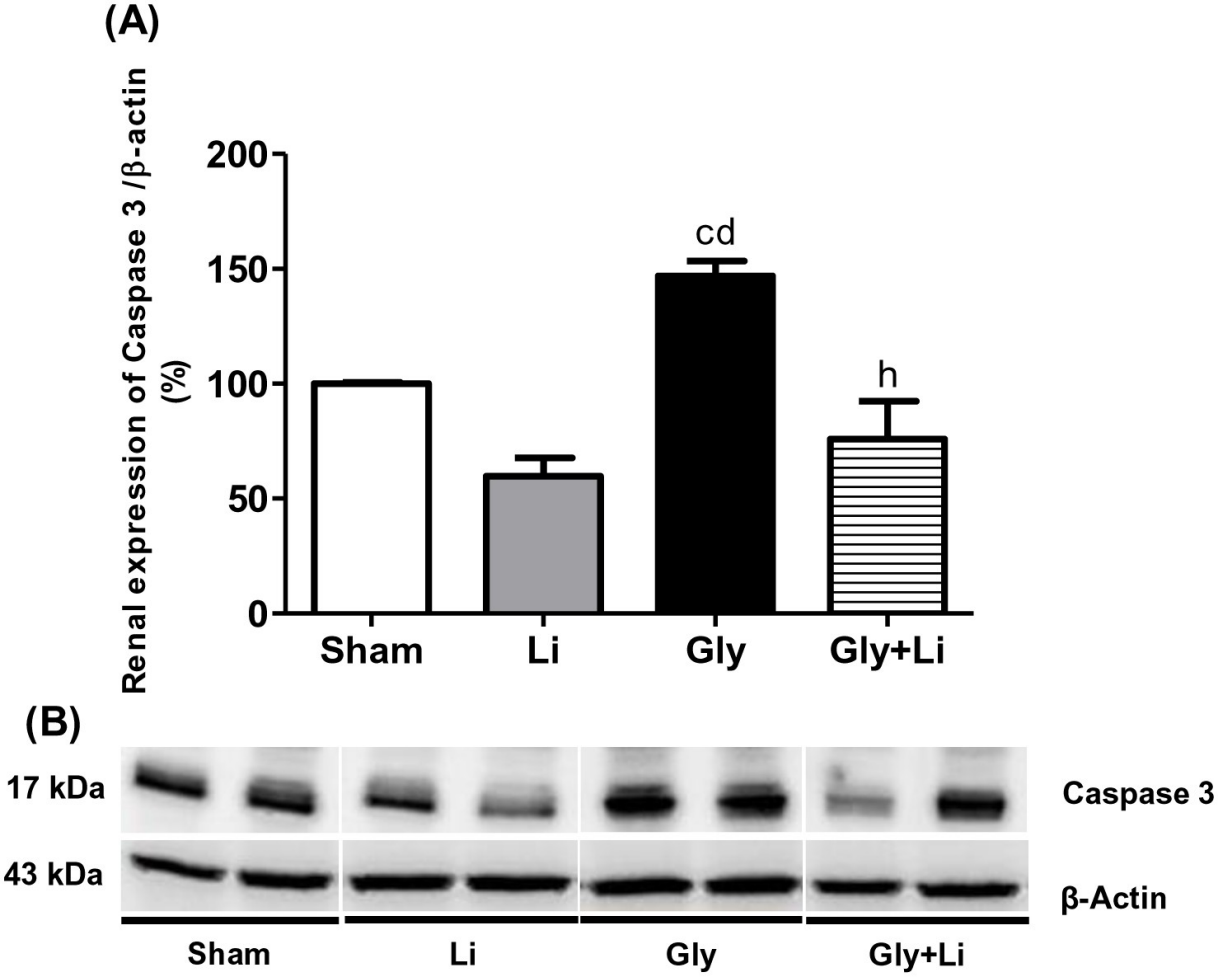
Semiquantitative immunoblotting for Caspase 3 expression in rat kidney tissue. (A) Caspase 3 densitometric analysis of kidney samples from Sham, Li, Gly and Gly+Li rats. (B) Representative immunoblots which reacted with anti-Caspase 3 revealing bands of 17kDa. Values are means ± SEM. ^c^p<0.05 vs. Sham; ^d^p<0.001 vs. Li, ^h^p<0.01 vs. Gly. Li, lithium; Gly, glycerol; Gly+Li, glycerol + lithium.

We evaluated the effect of a single dose of lithium on glycerol-induced cell apoptosis by Caspase 3 protein expression in renal tissue. Gly rats exhibited an increased Caspase 3 expression compared to Sham and Li rats. Treatment with lithium promoted a reduced renal expression of Caspase 3 in Gly+Li rats compared to Gly rats (Fig 5 and S2 Fig), indicating a protective effect of lithium on renal apoptosis. These results are consistent with the functional (Table 1) and histological data (Fig 1).

**Fig 6:**
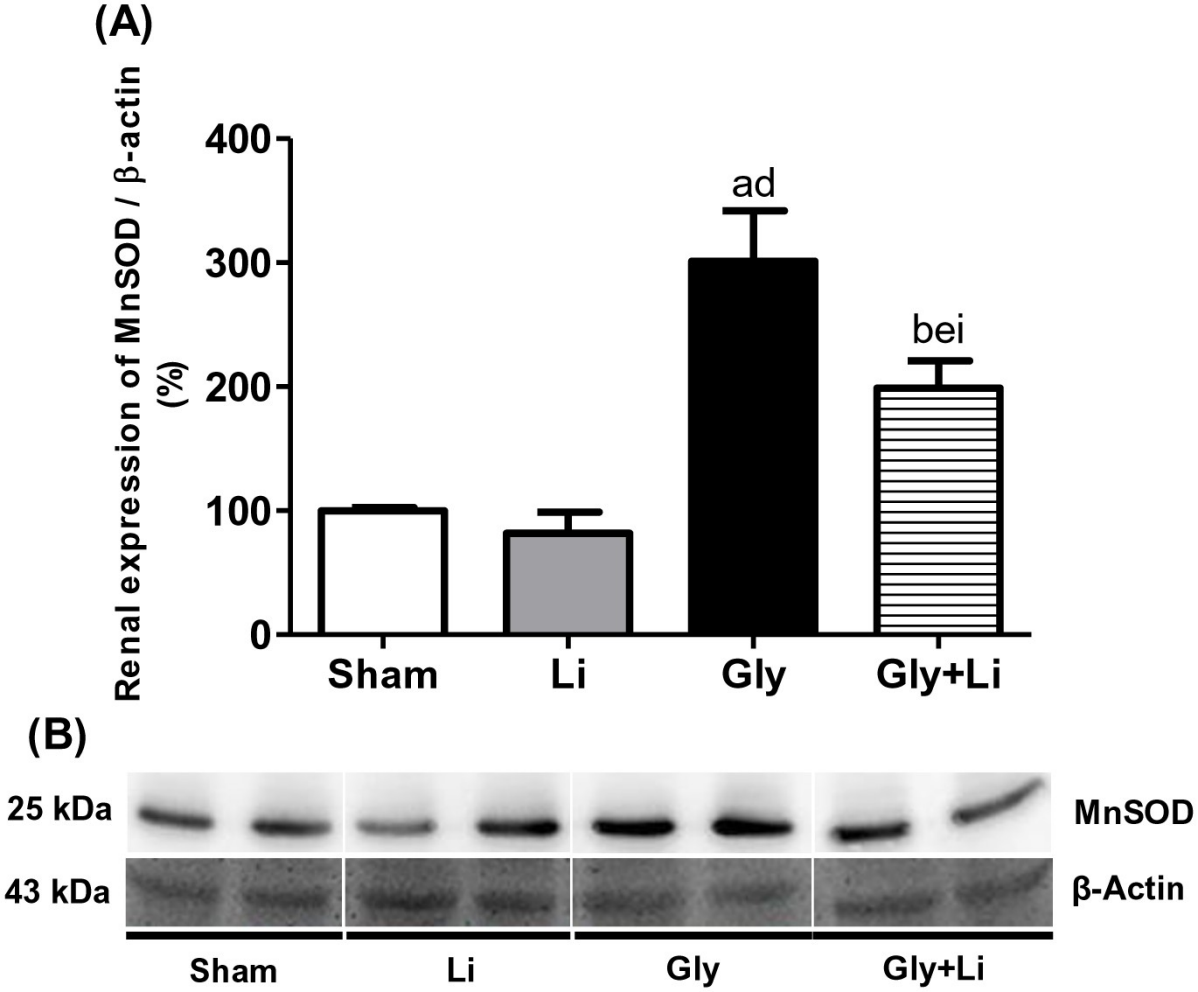
Semiquantitative immunoblotting for MnSOD expression in rat kidney tissue. (A) MnSOD densitometric analysis of samples from Sham, Li, Gly and Gly+Li rats. (B) Representative immunoblots which reacted with anti-MnSOD revealing a band of 25kDa. Values are means ± SEM. ^a^p < 0.001, ^b^p<0.01 vs. Sham; ^d^p < 0.001, ^e^p<0.01 vs Li; ^i^p < 0.05 vs. Gly. Li, lithium; Gly, glycerol; Gly+Li, glycerol + lithium.

Glycerol-induced AKI model is characterized by enhanced renal oxidative stress [22] and protective elements, such as MnSOD, may be straightly associated with mechanisms underlying the mitigation of AKI [23]. Gly group exhibited a higher MnSOD renal protein abundance compared to Sham and Li groups. Strikingly, lithium administration attenuated the renal expression of MnSOD in the Gly+Li group compared to the Gly group (Fig 6 and S3 Fig). Based on that, we could infer that lithium may play a role in the modulation of the redox state balance.

**Fig 7:**
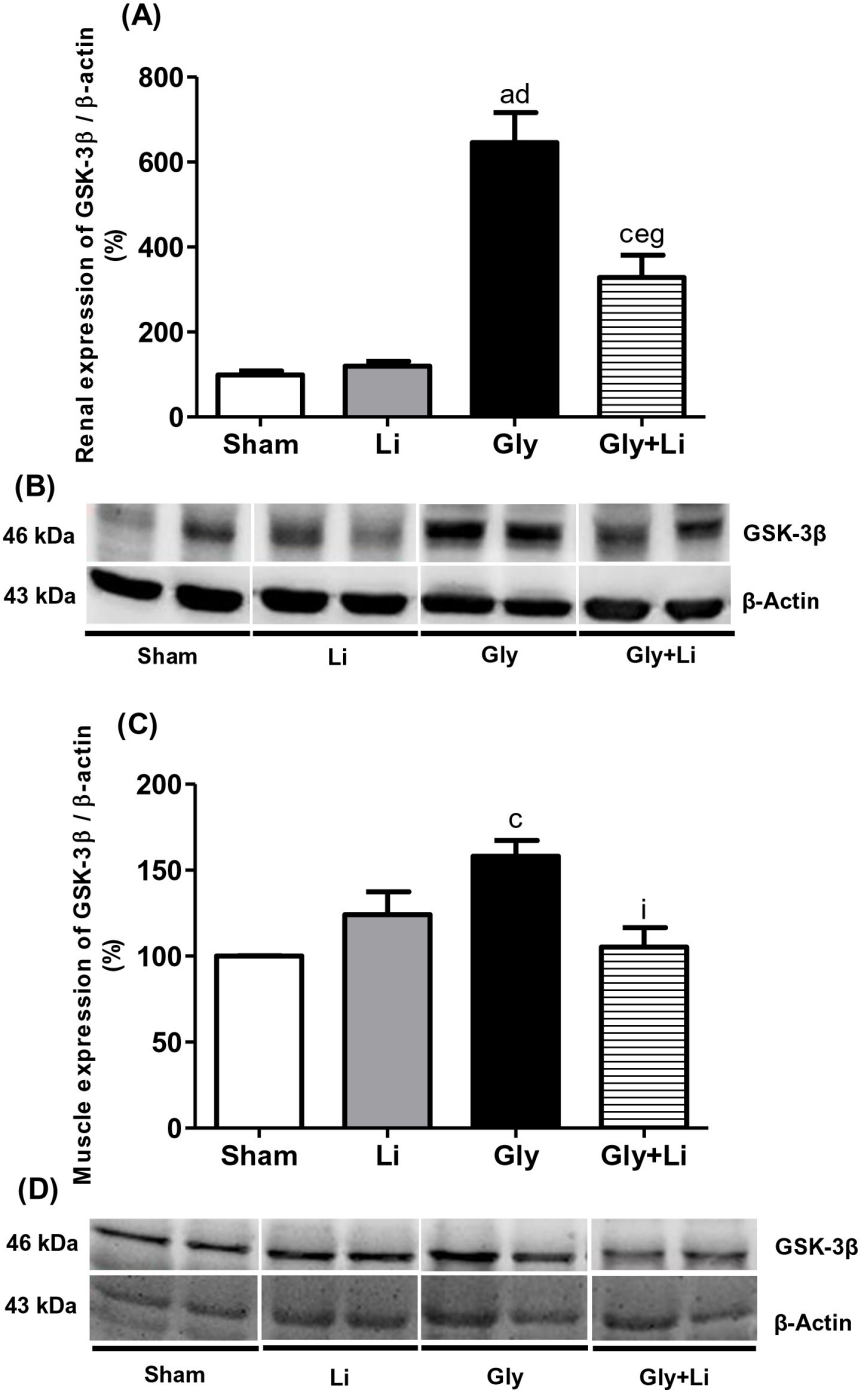
Semiquantitative immunoblotting for GSK-3β expression in rat kidney and muscle tissue. (A) GSK-3β densitometric analysis of kidney samples from Sham, Li, Gly and Gly+Li rats. (C) GSK-3β densitometric analysis of muscle samples from Sham, Li, Gly and Gly+Li rats. (B) and (D) Representative immunoblots which reacted with anti- GSK-3β revealing bands of 46kDa in kidney and muscle, respectively. Values are means ± SEM. ^a^p<0.001, ^c^p<0.05 vs. Sham; ^d^p<0.001, ^e^p<0.01 vs. Li; ^g^p<0.001, ^i^p<0.05 vs. Gly. Li, lithium; Gly, glycerol; Gly+Li, glycerol + lithium.

These renal/muscular therapeutic effects of lithium were associated with the inhibition of both renal and muscular GSK3β. Glycerol-injected animals showed a higher renal protein expression of GSK3β compared to Sham animals. Renal protein expression of GSK3β was attenuated in Gly+Li group, suggesting that inhibition of GSK3β may contribute to improve renal outcomes after rhabdomyolysis-associated AKI. Similarly, muscle GSK3β protein expression was increased in Gly animals compared to Sham animals and treatment with lithium promoted a reduction in that parameter in Gly+Li group compared to Gly group (Fig 7, S4 and S5 Figs). Our results indicate that inhibition of muscle protein expression of GSK3β could be related to accelerated muscular recovery.

## Discussion

Rhabdomyolysis-associated AKI following a single intramuscular injection of glycerol, equally distributed to both hind legs, is a validated model to investigate the mechanisms involved in this syndrome along with the study of effective therapeutic agents [4, 8, 14]. A previous study from our laboratory demonstrated a prophylactic and therapeutic protection effect of allopurinol in the same experimental model of rhabdomyolysis-associated AKI due to its actions on oxidative stress, apoptosis, inflammation and cell proliferation [14]. Along similar lines of study, Soares et. al reported that resveratrol protected against glycerol-induced renal injury by inhibiting lipid peroxidation and suppressing inflammatory signaling pathway [5]. Recently, some experimental studies and clinical trials showed that small doses of lithium are capable of ameliorate AKI and promote kidney repair [15, 24]. Lithium is the best-known GSK3β inhibitor and has been prescribed as the first line regimen to treat bipolar affective disorders [24]. However, multiple harmful off-target effects of lithium have been described since its approbation by Food and Drug Administration (FDA) in the 1970s. Severe kidney injury associated with long-term use and excessive serum concentrations of lithium is the most well documented and characterized side effect of this drug [25]. Renal adverse effects include nephrogenic diabetes insipidus and, sporadically, end stage renal disease. Notwithstanding the fact that mechanisms underlying lithium-induced renal pathologies are well understood in the literature, experimental studies revealed that short-term administration of lithium in lower amounts may attenuate several types of AKI [26]. In the present study, our glycerol-injected rats presented the characteristic pathogenic features of rhabdomyolysis-associated AKI such as impaired renal function, elevated plasma CPK levels, renal tubular injury, apoptosis, inflammation and oxidative stress. A single dose of lithium ameliorated renal dysfunction secondary to rhabdomyolysis probably due to its downregulation effect on muscle and kidney GSK3β protein expression.

Experimental studies suggest that rhabdomyolysis leads to a decrease in GFR probably due to impaired renal hemodynamics and morphology, such as intrarenal vasoconstriction, ischemic tubular injury, and tubular obstruction [9]. Nevertheless, the pathogenesis of glycerol-induced AKI is complex and still unclear [9, 27]. Gois et al. showed a reduction in GFR and RBF associated with an increase in RVR three hours after glycerol injection, however these levels returned to basal conditions after 24 hours. Those findings indicate that renal vasoconstriction is one of the key elements in the first hours of rhabdomyolysis-associated AKI pathogenesis [14]. Corroborating these data, several studies demonstrated that administration of glycerol led to a significant increase in both serum urea and creatinine 24 hours after glycerol injection [4, 11]. Similarly, our Gly animals presented a markedly GFR reduction compared to Sham animals. As expected, our Gly animals showed no changes in MAP, RBF and RVR compared to Sham animals 24 hours after glycerol injection, reaffirming that severe cortical ischemia occurs during the initial hours of glycerol-induced AKI and reflects on a poor long-term prognosis [28]. Treatment with a single dose of lithium recovered GFR in Gly+Li group, showing a protective effect of lithium in restoring renal function. In agreement with our results, Bao et al. reported that a low-dose lithium treatment promoted a kidney-protective effect and hastened renal function repair in animal models of AKI [15, 29].

In support of the aforementioned, our Gly group exhibited higher tubular injury score compared to Sham group. These results are consistent with previous studies which demonstrated that glycerol-induced AKI was associated with oxidative stress and lipid peroxidation of the proximal tubular cell, triggering the release of a series of pro-inflammatory mediators and leading to tubular necrosis [30]. Lithium administration attenuated renal damage in our Gly+Li group by ameliorating renal architecture after tubular necrosis. In accordance with our results, Alsady et al. reported that lithium treatment prevented acute tubular necrosis and lowered serum creatinine levels in nephrotoxicity-induced AKI models due to its stimulatory actions on tubular cell proliferation and repair [26]. The mechanisms by which lithium exerts a protective effect on kidney function has been recently attributed to the inhibition of GSK3β, a multitasking serine-threonine protein kinase involved in several pathophysiological processes [31, 32]. It is described that lithium increases the phosphorylation of a specific serine residue, Ser9, which is a hallmark of pharmacological GSK3β inhibition [33]. This inhibition promotes a reduction in the activity of GSK3β, resulting in a subsequent cell function alteration [34]. It is important to highlight that Ser9 phosphorylation does not exactly indicate cellular GSK3β activity itself [35], since other mechanisms may be involved in its activation [36]. Krishnankutty et al. reported that the increase in phosphorylated-Ser9 levels did not achieve complete GSK3β inactivation in cultured cells upon stimulation with growth factors [35]. Thus, Ser9 phosphorylation state determination is not enough and it is essential to evaluate actual GSK3β activity [36]. A few studies demonstrated that the potency of lithium effect may be also linked to the amount of magnesium, showing that Li+ act as uncompetitive inhibitors for the binding of the co-factor magnesium to GSK3 [37, 38]. GSK3β plays a pivotal role in AKI by promoting inflammation, oxidative stress and apoptosis which are considered the main mediators of renal dysfunction in glycerol-induced AKI [27, 32, 39]. Our study demonstrated that renal protein expression of GSK3β was augmented in Gly group and lithium treatment attenuated this parameter in Gly+Li group, indicating that the beneficial effects of lithium were probably due to the inhibition of renal GSK3β.

GSK3β is a key enzyme standing at the crossroads of several signaling pathways in different tissues such as skeletal muscle. Studies suggest that this enzyme may act as an endogenous inhibitor of protein synthesis and degradation, being responsible for the balance between anabolic and catabolic processes associated with protein breakdown [40]. Fang et al. showed that augmented protein degradation was related to higher GSK3β activity and reduced muscle amount of phosphorylated Ser9 in a murine model of burn injury [36]. They also reported that treatment with LiCl was able to reduce muscle protein breakdown in the same model described above [41].

Traditionally, CPK is a biomarker of muscle injury widely used for the assessment and management of rhabdomyolysis in order to prevent AKI. Although CPK is not straightly involved in the pathogenesis of rhabdomyolysis-associated AKI, CPK levels overabundance has been linked to the development of renal failure [42, 43]. McMahon et al. showed an association between higher initial CPK concentration and the risk of renal replacement therapy or in-hospital mortality in patients with rhabdomyolysis [44]. It is well known that CPK increases in rhabdomyolysis within 12 hours of the onset of muscle damage, peaks in approximately 1–3 days and decreases until the fifth day after muscle injury has ceased [45]. Our Gly animals exhibited augmented CPK levels and lithium treatment was effective in restoring this parameter in the Gly+Li animals. Moreover, GSK3β inhibition led to an overall enhancement in muscle fatigue resistance and mass recovery, improved muscle strength and reduced myotonia in different types of murine models of muscle wasting diseases, indicating that GSK3β has a crucial role in muscle regeneration [46–49]. A previous study from our laboratory showed higher CPK levels associated with significant muscular injury score 3 hours after glycerol injection [14]. Our results demonstrated that lithium administration recovered muscular protein expression of GSK3β in Gly+Li group. Therefore, these findings suggest that lithium protected the kidney from rhabdomyolysis-associated AKI. In addition, lithium treatment may have probably improved muscular damage since both plasma CPK and muscular protein expression of GSK3β returned to basal levels 24 hours after glycerol injection.

It is important to highlight that AKI is the most severe complication of rhabdomyolysis, which is the result of a wide variety of physical, thermal, toxic, metabolic, ischemic, infective and inflammatory muscle insults [14]. Intramuscular glycerol injection leads to rhabdomyolysis, followed by renal ischemia and myoglobinuria [11]. After severe muscular damage, several mechanisms could be responsible for the development of acute renal failure, such as 1- the release of myoglobin that is potentially nephrotoxic, 2- the volume depletion due to fluid transudation from the extracellular compartment to the injured tissue, and 3- the activation of systemic inflammatory response and vasoactive factors [50]. Moreover, myoglobin is reabsorbed by the proximal tubule cells and promotes oxidative stress, resulting in cortical acute tubular necrosis which is the hallmark of renal pathology in this model [11]. Thus, since higher CPK levels were associated with acute renal failure and the need for renal replacement therapy in patients [51], our findings suggest that therapeutic targeting of GSK3β with lithium treatment initially ameliorated rhabdomyolysis-induced AKI probably due to a decrease in CPK plasma levels.

Rhabdomyolysis-injured muscle cells release immunostimulatory molecules that reach renal tissue, where they activate NFκB signaling pathway [3]. Upregulation of the NFκB cascade plays an important role in the progression of glycerol-induced kidney injury through the synthesis of various pro-inflammatory mediators, such as macrophages and T lymphocytes, which have been positively correlated with impaired renal function and morphological changes [52]. In the present protocol, glycerol-injected animals exhibited an increased protein expression of p65 subunit of NFκB and a higher amount of macrophage infiltrate in renal tissue. Treatment with lithium ameliorated the inflammatory profile in the Gly+Li group. It is important to highlight that the determination of p65 subunit of NFκB renal protein expression does not necessarily correspond with the active phosphorylated form of this protein, however the increased number of macrophage cells observed in Gly group is sufficient to validate glycerol-induced AKI inflammatory effects [14]. Furthermore, GSK3β phosphorylates and activates the p65 subunit of NFκB and increases its transcriptional response resulting in inflammation [32]. Thus, our results allow us to infer that lithium may modulate renal inflammation due to the inhibition of GSK3β, leading to the suppression of NFκB and subsequent suppression of pro-inflammatory substances release.

In addition to impaired renal function and inflammation, oxidative stress has been linked to the development of myoglobin-induced renal injury in rhabdomyolysis-associated AKI [7]. Huerta-Alardín et. al reported that precipitation of heme protein can generate toxic free radicals and induce oxidative damage to the renal tubule [45]. MnSOD is the major antioxidant scavenger present in the mitochondria and may be straightly associated with mechanisms underlying the protection against AKI [11, 23]. Our Gly rats presented a higher MnSOD renal expression and a single dose of lithium attenuated this enzyme expression in the Gly+Li rats. Supporting our data, several studies have been demonstrating that glycerol-injected animals presented increased ROS levels in kidney homogenates and a higher 4-HNE tubular expression during the acute phase of rhabdomyolysis [16, 53]. In addition, Gois et al. showed a significant increase in plasma TBARS levels, as well as a higher MnSOD renal protein expression 3 and 24 hours after glycerol administration in a rhabdomyolysis-induced AKI model, respectively [14]. Moreover, despite the presence of oxidative stress, MnSOD renal protein expression might be upregulated due to the enhanced TBARS levels, indicating that redox state imbalance activated the mitochondrial enzymatic system in the damaged kidney [23]. Inhibition of GSK3 by lithium does not trigger antioxidant systems upon normal condition, but exacerbates its response under oxidative stress. Notwithstanding MnSOD is an antioxidant heat shock protein responsive to renal damage, Gly+Li rats showed a decrease in the renal expression of this element probably due to the antioxidant action of lithium and the recovery of kidney injury [23, 54].

Apoptosis is a vital process involved in several basal functions including cell turnover, cell differentiation and homeostasis. Inappropriate apoptosis leads to pathological conditions demonstrated in various models of AKI [55, 56]. Previous studies have shown that caspase-mediated apoptosis plays an important role in the pathogenesis of rhabdomyolysis-associated AKI [10, 56]. Caspase 3 cleaves intracellular proteins resulting in reduced proximal tubular cell adhesion, cell death and subsequent acute tubular necrosis [10, 57]. As expected, our Gly rats exhibited an enhanced renal protein expression of caspase 3. Treatment with lithium restored this cysteine-protease in our Gly+Li group, indicating that a single dose of lithium protected renal tubular cells and attenuated kidney damage. Supporting our data, Howard et al. demonstrated that GSK3β knockout mice presented lower levels of cleaved caspase 3 and Bax, as well as unchanged antiapoptotic protein Bcl-2 in a nephrotoxicity-induced AKI model. Therefore, GSK3β absence was probably responsible for the reduction in apoptosis and renal injury, improving survival after AKI [58]. Altogether, those findings uphold the efficacy of GSK3β inhibitors in the management of rhabdomyolysis-associated AKI through their anti-apoptotic effects.

In summary, a single-dose of lithium attenuated glycerol-induced AKI probably through its beneficial actions on inflammation, oxidative stress and apoptosis. This lithium protective effect on kidney may be attributed to the inhibition of GSK3β. Since rhabdomyolysis is a condition with high risk of evolution to acute kidney injury and efforts to minimize this risk include prompt treatment to prevent rhabdomyolysis-induced AKI, Homsi et al. demonstrated that progression to established renal failure could be totally avoided with prophylactic treatment in a retrospective analysis of patients with rhabdomyolysis admitted to an intensive care unit [50]. Moreover, a single-dose of lithium at a low dose has been showing to be safe with no record of damaging renal side effects [15, 26]. Thus, our study indicates that treatment with lithium could represent a possible therapeutic approach to mitigate rhabdomyolysis-associated AKI and could motivate new trials to determine the appropriate dosage for a better prognosis in patients who have experienced AKI followed by rhabdomyolysis.

## Supporting information

S1 Fig Original uncropped and unadjusted image for NFκB. Immunoblotting figures for NFκB expression of kidney samples from Sham, Li, Gly and Gly+Li rats.
(TIF)

S2 Fig Original uncropped and unadjusted image for Caspase 3. Immunoblotting figures for caspase 3 expression of kidney samples from Sham, Li, Gly and Gly+Li rats.
(TIF)

S3 Fig Original uncropped and unadjusted image for MnSOD. Immunoblotting figures for MnSOD expression of kidney samples from Sham, Li, Gly and Gly+Li rats.
(TIF)

S4 Fig Original uncropped and unadjusted image for GSK3β. Immunoblotting figures for GSK3β expression of kidney samples from Sham, Li, Gly and Gly+Li rats.
(TIF)

S5 Fig Original uncropped and unadjusted image for GSK3β. Immunoblotting figures for GSK3β expression of muscle samples from Sham, Li, Gly and Gly+Li rats.
(TIF)
