# Supplementary figures and images for: Administration of a single dose of lithium ameliorates rhabdomyolysis-associated acute kidney injury in rats
Source: PLoS One. 2023 Feb 16;18(2):e0281679. doi: 10.1371/journal.pone.0281679 (PMC9934413; doi:10.1371/journal.pone.0281679)

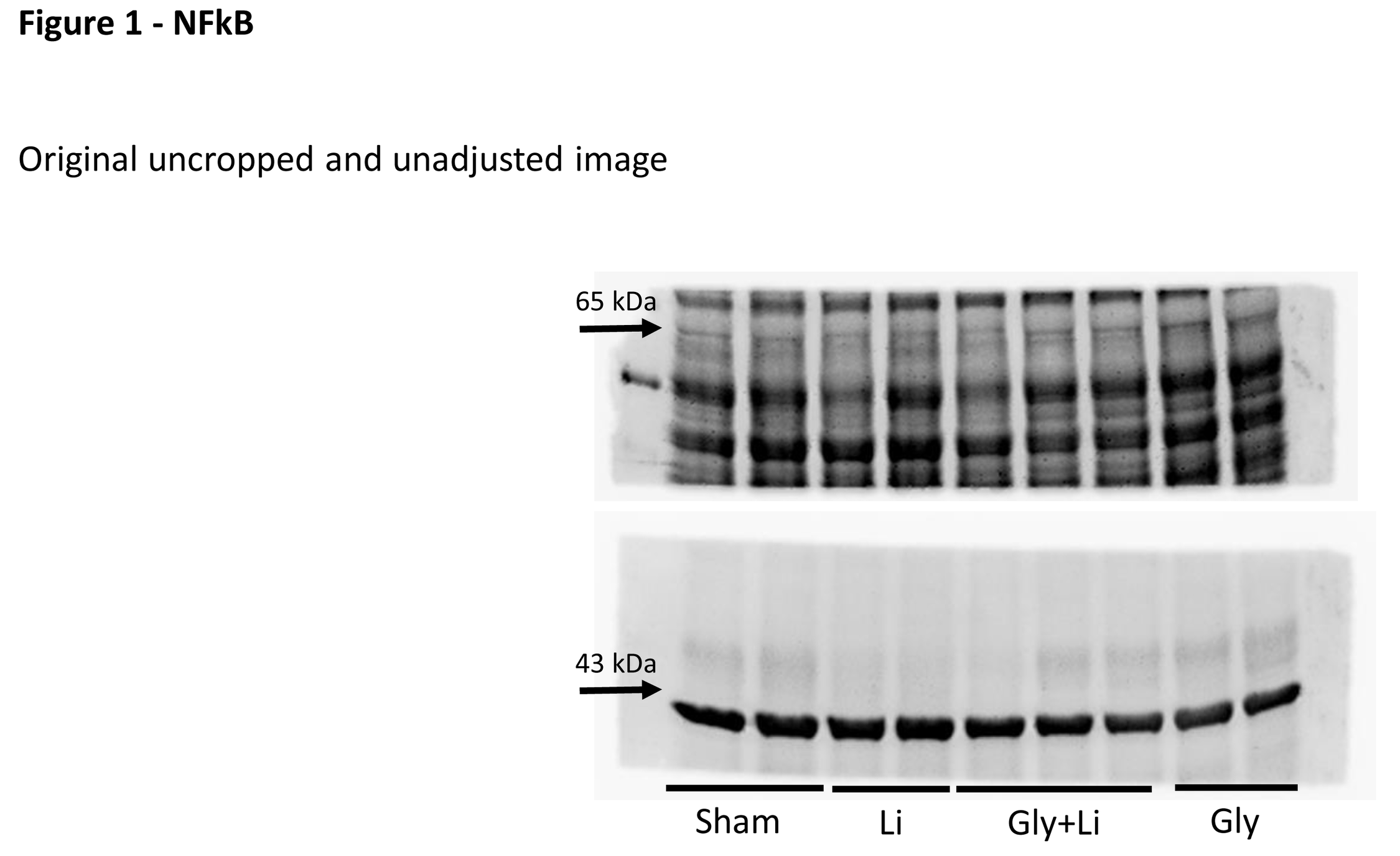

Supplement: S1 Fig — Immunoblotting figures for NFκB expression of kidney samples from Sham, Li, Gly and Gly+Li rats. (TIF) [file pone.0281679.s001.tif]

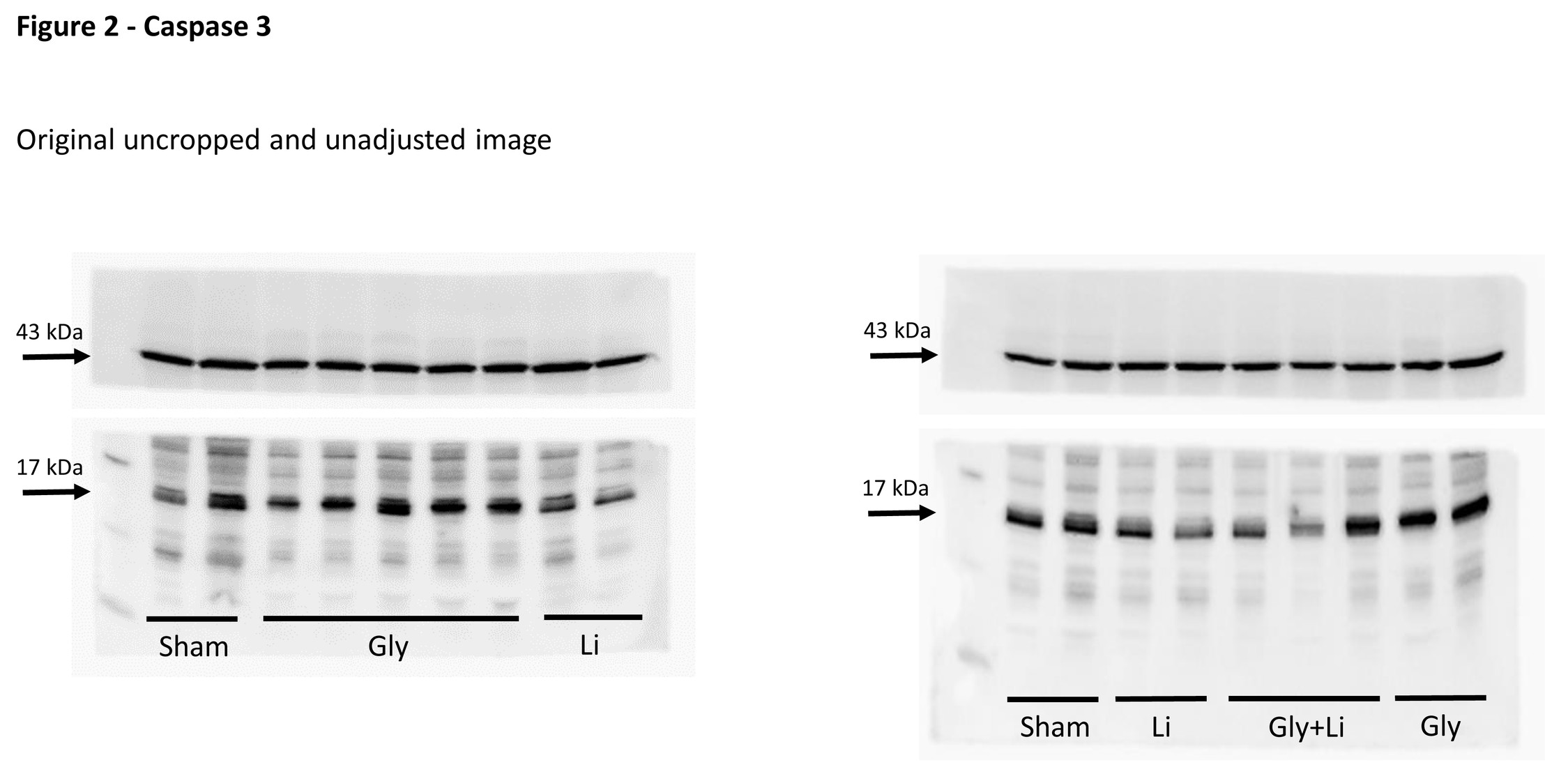

Supplement: S2 Fig — Immunoblotting figures for caspase 3 expression of kidney samples from Sham, Li, Gly and Gly+Li rats. (TIF) [file pone.0281679.s002.tif]

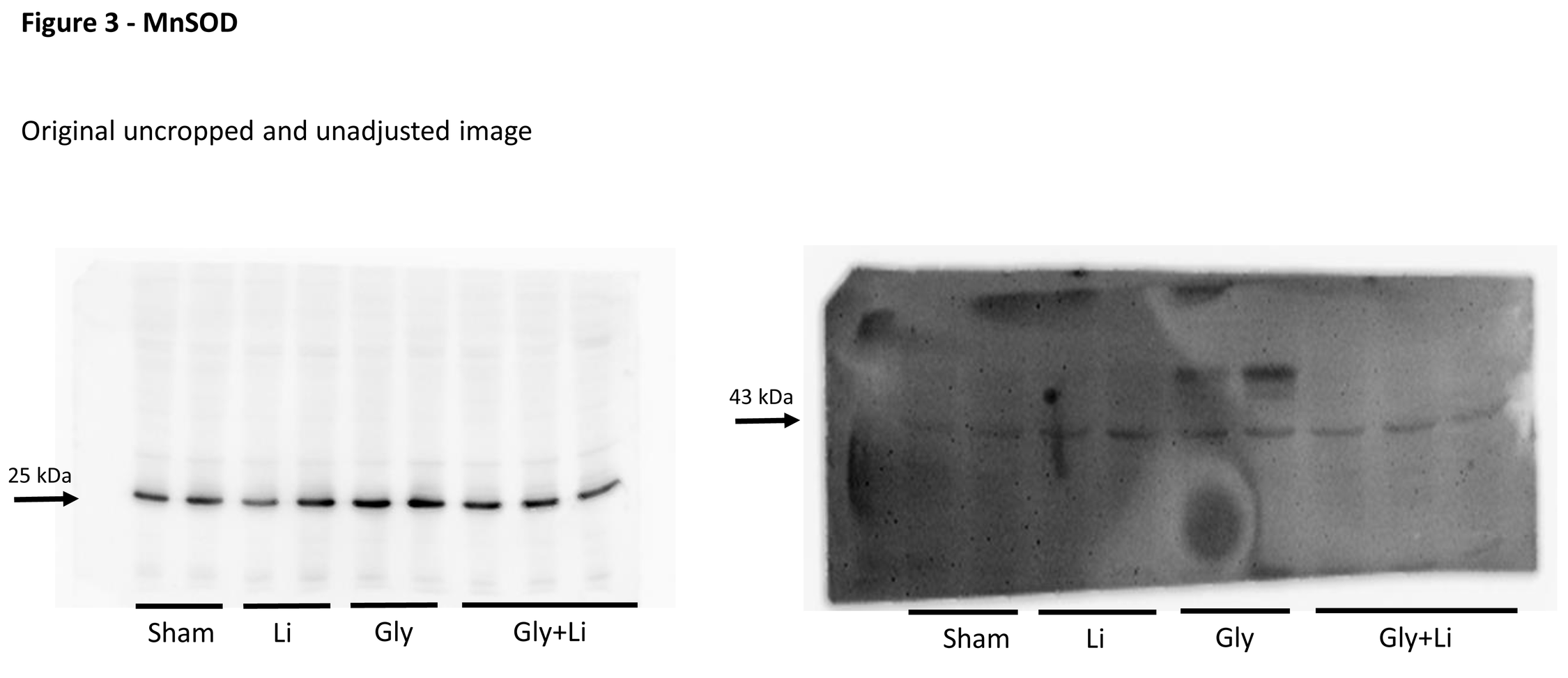

Supplement: S3 Fig — Immunoblotting figures for MnSOD expression of kidney samples from Sham, Li, Gly and Gly+Li rats. (TIF) [file pone.0281679.s003.tif]

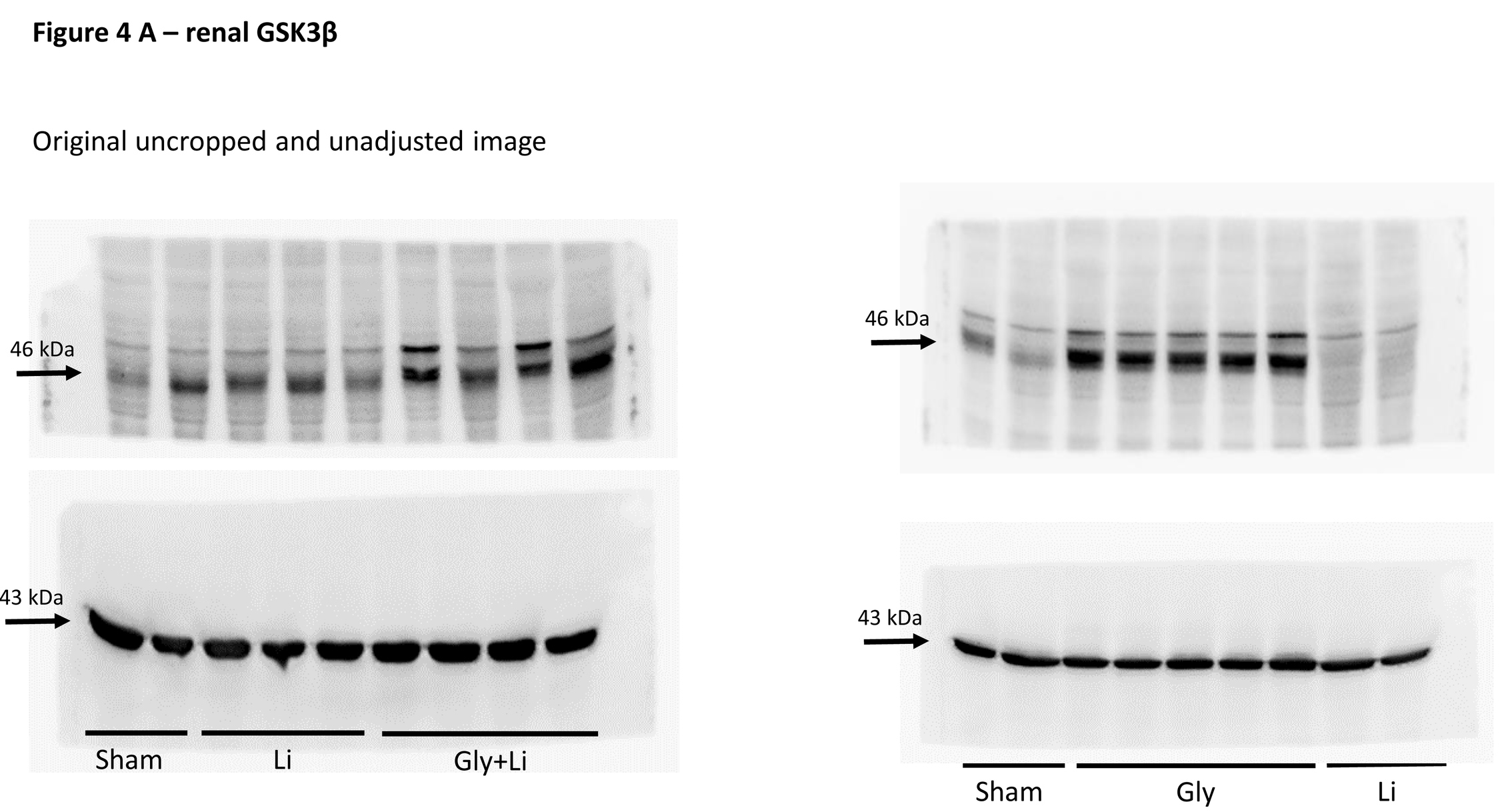

Supplement: S4 Fig — Immunoblotting figures for GSK3β expression of kidney samples from Sham, Li, Gly and Gly+Li rats. (TIF) [file pone.0281679.s004.tif]

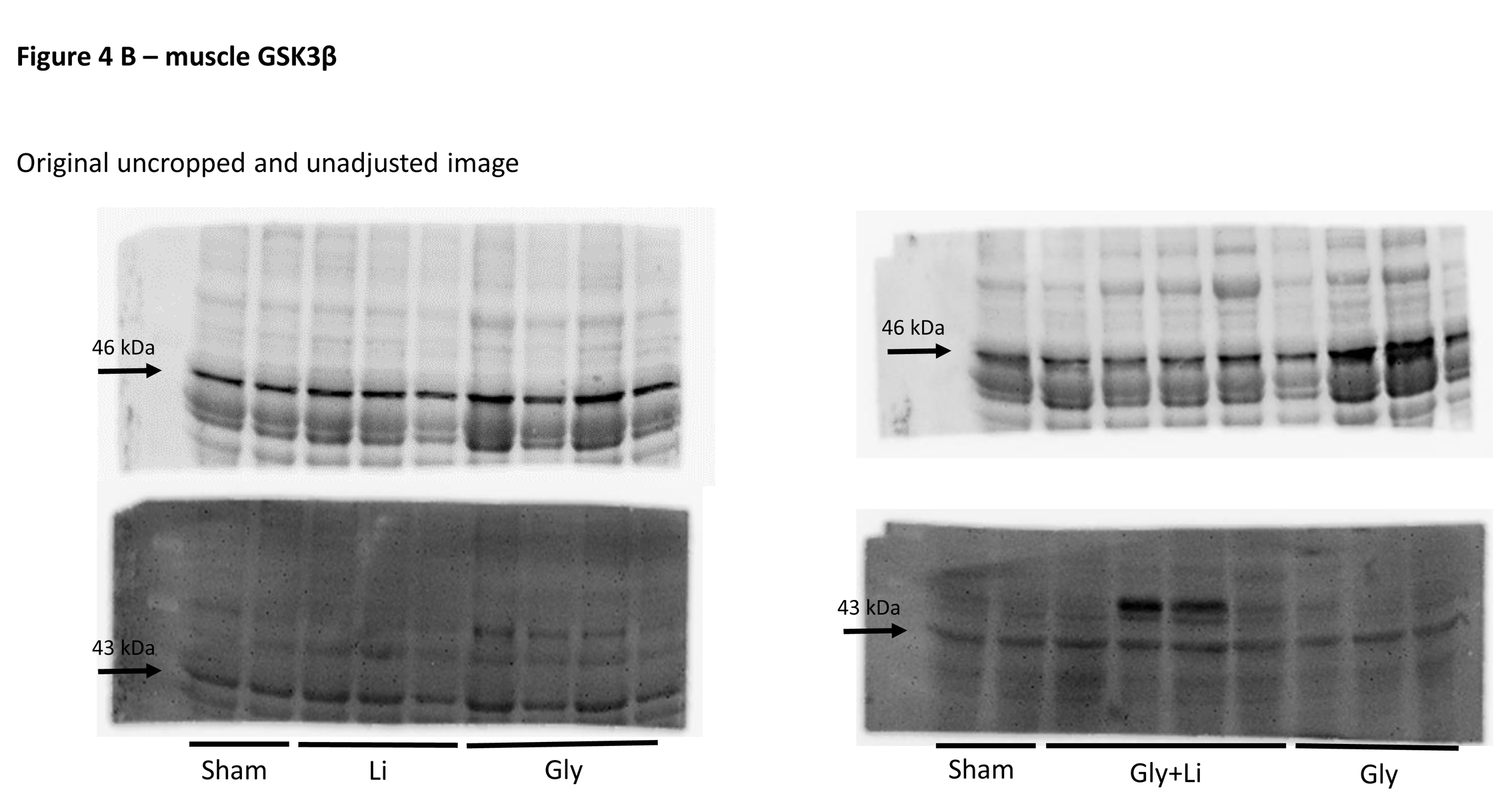

Supplement: S5 Fig — Immunoblotting figures for GSK3β expression of muscle samples from Sham, Li, Gly and Gly+Li rats. (TIF) [file pone.0281679.s005.tif]
